# Case report: A rare DLST mutation in patient with metastatic pheochromocytoma: clinical implications and management challenges

**DOI:** 10.3389/fonc.2024.1394552

**Published:** 2024-05-21

**Authors:** Chang Li, Liang Han, Yuming Song, Rui Liu

**Affiliations:** ^1^Department of VIP Unit, China-Japan Union Hospital of Jilin University, Changchun, Jilin, China; ^2^Department of Pathology, China-Japan Union Hospital of Jilin University, Changchun, Jilin, China

**Keywords:** pheochromocytoma, neoplasm metastasis, exome sequencing, dihydrolipoamide succinyltransferase, case report

## Abstract

**Background:**

Pheochromocytoma is one of the most hereditary human tumors with at least 20 susceptible genes undergoing germline and somatic mutations, and other mutations less than 1% -2%. In recent years, other rare mutations have gradually been discovered to be possibly related to the pathogenesis and metastasis of pheochromocytoma. Most patients with pheochromocytoma experience common symptoms like headaches, palpitations, and sweating, while some may have less common symptoms. The diversity of symptoms, genetic mutations, and limited treatment options make management challenging.

**Case presentation:**

A 53-year-old woman was hospitalized after experiencing episodic epigastric pain for one month. A mass was found in her right adrenal gland and she underwent robot-assisted laparoscopic surgery, revealing a pheochromocytoma. At the 16-month follow-up, multiple metastatic lesions consistent with metastatic pheochromocytoma were found. A germline mutation in the dihydrolipoamide succinyltransferase (DLST) gene (c.330 + 14A>G) was detected, and despite trying chemotherapy and adjuvant therapy, the patient had a limited response with an overall survival of 27 months.

**Conclusions:**

DLST mutation is one of the rare pheochromocytoma-related mutated genes, and genetic sequencing is crucial for effective clinical management.

## 1 Introduction

Pheochromocytoma (PCC) and paraganglioma (PGL) are collectively known as PPGL (1). Clinical manifestations of hypertension are present in over 90% of patients, while symptoms such as headache, palpitations, and sweating are reported in more than 50% of cases. PPGL has been categorized into metastatic and non-metastatic forms (1). 10% of PCC and 15–35% of PGL are malignant but metastatic diseases are rare (2, 3). PPGL is recognized as one of the most heritable tumors among all human malignancies, with genetic factors accounting for approximately 40% (4). The activation of susceptibility genes in key pathways, including pseudohypoxia, kinase, and Wnt signaling, are identified (5–8). Tumors classified within Cluster 1 typically exhibit a noradrenergic biochemical phenotype and are associated with a heightened risk of sustained hypertension. Gene sequencing, metaphranes, and abdominal imaging are useful for diagnosing PCC (9). Surgery is the preferred treatment, but systemic options are available for inoperable cases with limited effectiveness. Patients with mutation should receive personalized lifelong monitoring (10).

A case study of a female with PCC metastasis occurring 16 months post-surgery was presented, characterized by atypical clinical manifestations. Whole-exome sequencing revealed DLST gene mutations, suggesting a potential association with PCC development. A comprehensive review of literature was conducted to discuss the clinical management.

## 2 Case presentation

### 2.1 Medical history and preoperative examination

**Figure 1:**
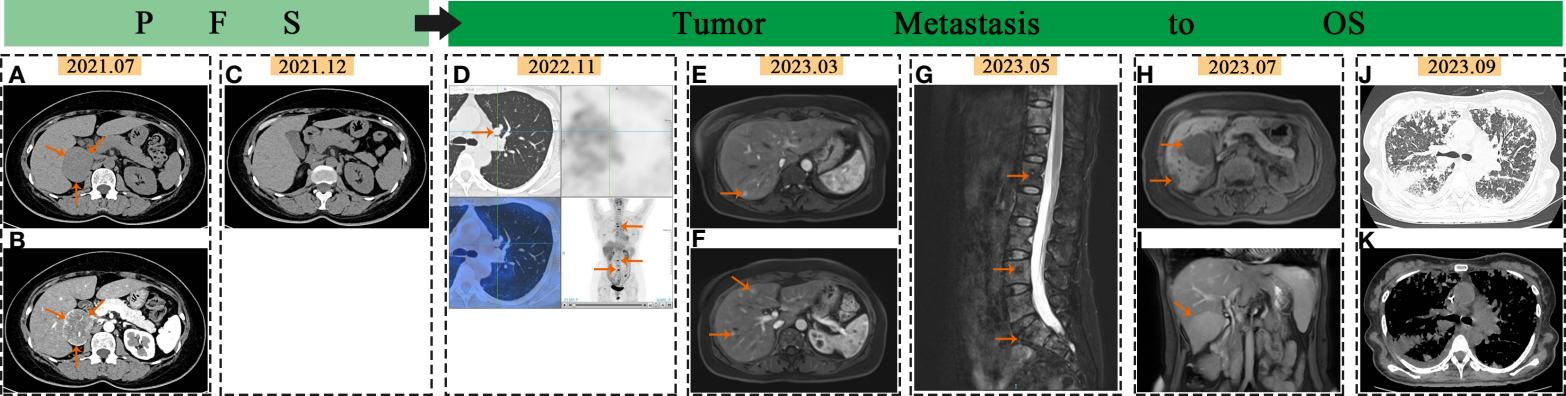
Imaging changes in PCC: In July 2021, the arrows pointed to the adrenal mass on abdominal and enhanced CT scans **(A, B)**. In December 2021, no abnormalities were seen in abdominal CT scans **(C)**. In November 2022, the arrows pointed to lung and bone metastasis on PET-CT **(D)**. In March 2023, the arrows indicated liver metastases on liver MR images **(E, F)**. In May 2023, the arrows pointed to spine metastases on MR images **(G)**. In July 2023, the arrows indicated liver metastases on liver MR images **(H, I)**. In September 2023, CT scan showed extensive lung metastases with inflammation **(J, K)**.

A 53-year-old female without prior medical history presented with episodic upper abdominal pain in June 2021.The patient exhibited no symptoms of paroxysmal or persistent hypertension, headache, palpitations, sweating, vision loss, body weight loss or other related manifestations. No retinal hemangioma was detected. In July 2021, the patient exhibited normal respiration, heart rate (80 beats/min), and blood pressure (130/80mmHg), with no abnormal findings noted during cardiopulmonary and abdominal examinations. Adrenal enhancement computed tomography (CT) revealed a space occupying lesion in the right adrenal region (about 4.2×5.9cm in size) (**Figures 1A, B**) and normal lung CT. 18F-fuorodeoxyglucose positron emission tomography (PET)/CT revealed a mass in the right adrenal region with unevenly increased glucose metabolism (maximum cross-sectional area of 5.68×4.81 cm). Auxiliary examinations of the central nervous system, heart, kidney, and pancreas revealed no abnormalities. Metanephrine (MN) and neuron specific enolase (NSE) showed no abnormalities. Blood normetanephrine (NMN) was about three times than the normal upper limit (**Supplementary Table 1**). CgA concentration in blood and fractionated metanephrines in 24 hour urine weren’t conducted. The patient’s blood routine, liver and kidney function, blood glucose levels, urinary occult blood, and other biochemical indicators showed no significant abnormalities. Normal ACTH rhythm, cortisol rhythm, and renin activity were observed (**Supplementary Table 4**). Aldosterone levels in the supine position were measured at 219.4pg/ml (reference range 10.0–160.0pg/ml), while orthostatic aldosterone levels were recorded at 415.5pg/ml (reference range 40–310pg/ml).

### 2.2 Surgical treatment and postoperative pathology

**Figure 2:**
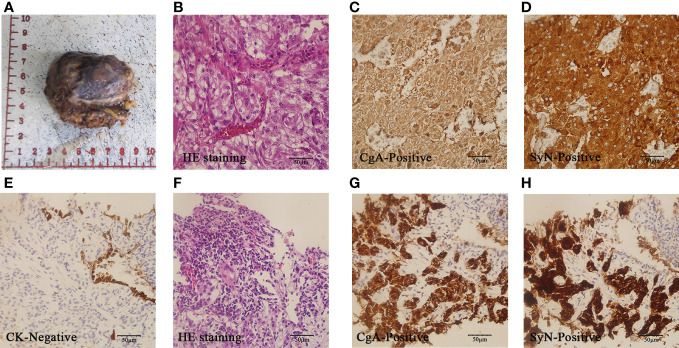
Pathology of PCC after surgery and metastasis:A-D showed pathology images of PCC after surgery. The gross specimen was a right retroperitoneal adrenal mass with incomplete capsule **(A)**. HE staining revealed small nests of chief cells with small and interspersed blood vessels(× 400) **(B)**. Tumor cells demonstrated diffuse CgA positivity (× 400) **(C)** and SyN positivity(×400) **(D)**. **(E–H)** displayed pathology images of lung metastases. Lung metastatic cells were negative for CK (× 400) **(E)**. The HE staining showed similar characteristics to the primary tumor (× 400) **(F)**. The lung metastatic cells exhibited diffuse CgA positivity (× 400) **(G)** and SyN positive (× 400) **(H)**.

To mitigate the risk of cardiovascular complications, the patient was administered oral doxazosin in July 2021, followed by oral bisoprolol prior to surgery. The planned procedures include laparoscopic retroperitoneal lesion resection, right adrenal mass resection, and abdominal adhesiolysis. Intraoperatively, the presence of greater omentum tissue at the inferior margin of the liver and intestinal adhesions was noted. Following dissociation, a mass measuring approximately 5.0 x 5.0 cm, exhibiting moderate activity and in close proximity to the right adrenal gland, was identified. No discernible abnormalities were detected in the liver, stomach, colon, or small intestine. Based on preoperative evaluations and intraoperative observations, the surgical approach was maintained without alterations. The surrounding tissue of the tumor was found to be fully free, allowing for the complete removal of the tumor. Postoperative pathology revealed a retroperitoneal adrenal tumor measuring 5.7 x 4.5 x 4cm and weighing 75g, with incomplete capsular involvement. Histological examination revealed the presence of small nests of chief cells accompanied by small and interspersed blood vessels. Immunohistochemical (IHC) staining indicated positivity for chromogranin A (CgA), synaptophysin (SyN), Ki-67 (5%+), succinate dehydrogenase B (SDHB), NSE, and sustentacular cells (individual cells+) (**Figures 2A–D**). The patient presented with atypical symptoms and signs of PCC. Based on qualitative diagnostic criteria (elevated blood NMN levels exceeding twice the upper limit of normal) and localization diagnostic methods (adrenal CT, PETCT) conducted prior to surgery, PCC was highly suspected. GAPP score was 5. The final clinical diagnosis was PCC (T3N0M0 stageIII).

### 2.3 Metastasis and postoperative treatment

The patient remained asymptomatic during routine postoperative follow-up. The adrenal CT was normal 5 months after operation (**Figure 1C**). Levels of MN, NMN, and NSE were within normal limits. 16 months post-operation,18F-FDG PET/CT imaging revealed increased glucose metabolism in the bilateral lungs and subpleural region. Additionally, heightened glucose metabolism was observed in the sternum, cervical 2, thoracic 4, 5, 7, 8, lumbar 3**–**5 vertebral bodies, left iliac bone, and right femur (**Figure 1D**). Lung CT showed multiple metastases. Further imaging with Gallium-68 labeled 1,4,7,10-tetraazacyclododecane-1,4,7,10-tetraacetic acid-N-octyl-D-phenylalanine and 18F-Dihydroxyphenylalanine PET/CT demonstrated widespread bone metastases, multiple lung metastases with high expression of growth inhibitory receptors, and liver metastases in both lobes with low expression of growth inhibitory receptors. The lung and bone lesions showed positive results for CgA, SyN, SDHB, vimentin (VIM), CD56, GATA3, and somatostatin receptor 2 (SSTR 2), with Ki-67 levels at 30% in the lung and 5% in the bone, indicating metastatic PCC (**Figures 2E–H**). Levels of MN, NMN,catecholamine (CA) and NSE were still within normal range.

Whole Exome Sequencing Analysis Reveals: A point mutation in the DLST Gene (c.330 + 14A>G), potentially linked to PGL Type 7, and a mutation in the Cyclin D1 (CCND1) Gene (c.575**–**13C>T), potentially associated with Von Hippel-Lindau Syndrome(**Supplementary Table 2**). The patient underwent pre-treatment evaluation and preparation for systemic therapy of metastatic PCC. The evident adverse reactions of nausea and vomiting were observed in patients receiving 68Ga-dotatate PET/CT. Taking into consideration the patient’s physical tolerance, the ultimate treatment regimen consisted of oral temozolomide (300mg administered once daily for 1**–**5 days, with a treatment course repeated every 28 days) and subcutaneous denosumab (120mg administered once every 4 weeks). Following the completion of one course of oral temozolomide, treatment was discontinued due to notable gastrointestinal symptoms. Subsequent treatment included continuation of the denosumab regimen alongside subcutaneous injections of octreotide acetate microspheres (60mg administered once every 4 weeks), interspersed with traditional Chinese medicine anti-tumor therapy. Subsequent to treatment initiation, the frequency of follow-up assessments was escalated. Blood parameter monitoring was conducted based on clinical status, with imaging evaluations scheduled approximately every two months. The imaging changes of metastatic lesions in March and May 2023 are shown in **Figures 1E–G**. By July 2023, the patient began experiencing significant dyspnea and intermittent hemoptysis, indicating rapid disease progression (**Figures 1H, I**). The adverse bleeding reaction associated with tyrosine kinase inhibitors (TKIs) treatment poses a limitation on the utilization of these drugs by the patient. In September 2023, she was hospitalized for worsening breathing difficulties, vomiting, and anemia. Metastatic lesions in the liver and lungs had increased (**Figures 1J, K**), along with elevated levels of NMN, NE, and NSE(**Supplementary Table 1**). She was diagnosed with advanced stage IV PCC. Palliative treatment was provided as she was in the terminal stage of the tumor. **Supplementary Table 3** showed the detailed timeline, symptoms and the treatment process.

## 3 Discussion

PPGL have a poor prognosis and limited treatment options. The key to diagnosis lies in appropriate biochemical tests and molecular IHC (11). Approximately 40% of PPGL cases are associated with germline mutations, making genetic testing crucial for early detection of genetic syndromes, follow-up of high-risk patients, and guidance of treatment (12). Half of the mutated genes in PPGL are members of the tricarboxylic acid (TCA) cycle. Recent studies have identified DLST as a component of the rate-limiting enzyme of the TCA cycle, and disruption of DLST has been linked to pseudohypoxia, which contributes to the occurrence and progression of PPGL (13). However, the reported cases are limited in number, and there is lack of comprehensive clinical data. Our patient exhibited DLST point mutations, PCC characterized by atypical clinical symptoms but high malignancy, multiple site metastasis, and a suboptimal response to treatment.

### 3.1 PCC gene sequencing

There has been a growing recognition of asymptomatic cases of PPGL through familial and germline mutation testing in recent years (9). About 15–17% of patients with PPGL will develop metastasis (14). The natural course of metastatic PPGL is highly heterogeneous, with 5-year survival rates ranging from 40% to 85% (15, 16). Stage IV PPGL has a significantly shorter overall survival (OS) (median OS 8.8 years) compared to stages I-III (17). Current guidelines recommend a comprehensive approach involving simultaneous localization diagnosis, qualitative diagnosis, and genetic counseling to accurately diagnose PCC/PGL (18). PPGL is linked to mutations in 20+ genes, categorized into three groups by TCGA: pseudohypoxia (cluster 1), kinase signaling (cluster 2), and Wnt signaling (cluster 3) (5–8). These mutations cause metabolic and epigenetic imbalances, promoting tumor invasiveness and metastasis (8). Our review focuses on PPGL patients with mutations in these clusters (**Table 1**).

**Table 1:** Clinical features of PPGL with mutations in susceptibility genes.

Gene	Mutation Frequency%	Gene Type	Tumor Type	Syndrome/Other Tumors	Family History%	Metastatic Risk%	References
Cluster 1 A (Krebs cycle)							
SDHD	7–10	AD, maternal imprinting	HNPGL>>PGL/PCC	PGL1/GIST, RCC, PA	40–50	1–9	(19)
SDHB	8–10	AD	ATPGL>>HNPGL/PCC	PGL4/GIST, RCC, PA, TT	10–24	25–50	(20)
SDHA	<5	AD	PCC/PGL	PGL5/GIST, RCC, PA	<10	Rare	(21)
SDHAF2	<1	AD, maternal imprinting	HNPGL>PCC	PGL2	>50	Rare	(22)
SDHC	1	AD	HNPGL>PCC/PGL	PGL3/GIST, RCC	<50	Rare	(23)
FH	<5	AD	PCC/PGL	Leiomyoma, RCC	Not obvious	>50	(24)
MDH2	<1	AD	PCC/ATPGL	NA	Not obvious	40	(25)
IDHx	<1	GM/SM	PGL	Brain tumor glioblastoma, AML	NA	NA	(26)
SLC25A11	<1	GM	PGL	NA	Not obvious	High?	(27)
GOT2	<1	GM	ATPGL	NA	NA	NA	(28)
DNMT3A	<1	GM	PGL	AML	NA	NA	(29)
DLST	<1	GM	PCC/PGL	NA	NA	Rare	(13)
Cluster 1B Hypoxia signaling							
VHL	5–10	AD	PCC>>PGL	VHL	25–50	1–9	(30)
EPAS1	1	SM>GM	PCC/ATPGL	Pacak-Zhuang syndrome/Hereditary erythrocytosis, Somatostatinoma	NA	Rare	(31)
EGLN1/EGLN2	<1	GM	PCC/ATPGL	Erythrocytosis	NA	Rare	(32)
Cluster 2							
RET	5	AD	PCC	MEN2 syndrome	25–50	<1	(33)
NF1	3–30	SM>GM	PCC	NF1syndrome	10–24	1–9	(34)
TMEM127	<2	AD	PCC>PGL	RCC	Rare	15	(35)
MAX	1	AD	PCC>PGL	Renal oncocytoma	25–50	Rare	(36)
H-RAS	7	GM/SM	PCC	NA	NA	NA	(37)
KIF1B	<1	GM	PCC	Neuroblastoma	NA	NA	(38)
MEN1	<1	GM	PCC/HNPGL	MEN1syndrome	NA	NA	(39)
Cluster 3							
MAML3	7	Fusion genes	PCC/PGL	Neuroblastoma	NA	High?	(40)
CSDE1	<1	SM	PCC/PGL	NA	NA	NA	(4)

AD, Autosomal dominant inheritance; GM, Germline mutation; SM, Somatic mutation; HNPGL, head and neck paraganglioma; GIST, gastrointestinal stromal tumor; RCC, pheochromocytoma; PA, pituitary adenoma; ATPGL, abdominal or thoracic paraganglioma; TT, Thyroid tumor; NA, Not Available.

In recent years, DLST mutations, accounting for less than 1% of cases, have also been identified as contributing to PPGL. A review of cases reported in the literature on DLST mutations reveals that only three authors have reported a total of 12 patients (**Table 2**). In 2019, Remacha et al. have described a new PPGL susceptibility gene DLST, which encodes the dihydrolipoamide S-succinyltransferase (13). The dihydrothiamide S-succinyltransferase encoded by the DLST gene is a rate limiting enzyme in the Krebs cycle of cluster 1 subgroup (43). Additionally, Alexandre et al.’s study indicated that DLST likely pathogenic variants may confer susceptibility to PPGL, with a predicted low penetrance (41). The DLST germline variant (p.gly374glu) can cause functional impairment and promote tumorigenesis by increasing α-ketoglutarate levels and activating the pseudohypoxic pathway (13). These patients exhibit sporadic occurrences, with NM abnormalities being more prevalent, and are more likely to develop chest and abdominal PPGL (41). Our case identified a mutation near the splice site of the DLST gene, which may lead to abnormal protein synthesis. The patient did not show typical symptoms of PCC, but NM levels were elevated before surgery and during metastasis, consistent with PCC caused by DLST mutation. There was no evidence of CCND1 gene mutations in the pathogenesis of PCC. The absence of consistent clinical manifestations and test results throughout the disease process of von Hippel Lindau syndrome due to CCND1 gene mutations suggests that the correlation between this specific mutation and the onset of pheochromocytoma was not present in this case (43). The patient’s lack of VHL gene mutations and absence of literature on VHL and DLST dual mutations were noted. Unfortunately, further genetic sequencing and epigenetic analysis of the patient’s family were unsuccessful.

**Table 2:** Clinical data of DLST mutations in PPGL patients.

Year	Gender	Age	Tumor Type	Other tumors	Metastasis	Biochemical phenotype	cDNA variants	Protein changes	Prediction	LOH
Remacha et al., 2019 (13)	male	45	PCC	─	yes	NM	c.692G>A	p.Arg231Gln	deleterious	no
Remacha et al., 2019	female	63	HNPGL	─	no	NS	c.910G>A	p.Asp304Asn	neutral	NA
Remacha et al., 2019	female	27	ATPGL	UEC	no	NM	c.1121G>A	p.Gly374Glu	deleterious	yes
Remacha et al., 2019	male	38	ATPGL	─	no	NM	c.1121G>A	p.Gly374Glu	deleterious	yes
Remacha et al., 2019	female	24	ATPGL and PCC	─	no	NM	c.1121G>A	p.Gly374Glu	deleterious	yes
Remacha et al., 2019	male	29	ATPGL	─	no	NM	c.1121G>A	p.Gly374Glu	deleterious	NA
Remacha et al., 2019	male	29	ATPGL	─	no	NM	c.1265A>G	p.Tyr422Cys	deleterious	NA
Remacha et al., 2019	male	54	PCC	PA	no	NM	c.1060–3T>A	─	─	no
Buffet et al., 2021 (41)	male	23	ATPGL	─	no	CA	c.1151C>T	p.Pro384Leu	probably damaging	yes
Buffet et al., 2021^a^	male	71	PCC and ATPGL	─	no	NM	c.1121G>A	p.Gly374Glu	deleterious	no
Mellid et al., 2023^b^ (42)	male	56	bilateral PCC	MTC,NF1syndrome	no	NM	NA	p.Gly374Glu	deleterious	no
Mellid et al., 2023^c^	female	55	PCC	─	no	M>NM	NA	p.Gly374Glu	deleterious	no

LOH, Loss of heterozygosity; PCC, pheochromocytoma; PGL, paraganglioma; HNPGL, head and neck paraganglioma; ATPGL, abdominal or thoracic paraganglioma; NM, normetanephrine; NS, non-secretory; CA, catecholamine; M, metanephrine; NA, not available; UPD, uniparental disomy; PA, pituitary adenoma; MTC, medullary thyroid carcinoma; NF1,neurofibromatosis type 1;a,with DLST somatic mutation; b, with NF1 germline mutation; c, with NF1 somatic mutation. “-” means no.

### 3.2 Symptoms, test results, and treatment for PCC

The primary symptom of PPGL is persistent or paroxysmal hypertension with target tissue damage (44). Clinical symptoms can vary from no symptoms to life-threatening events, even with normal blood pressure (45). Our patient initially had mild abdominal pain. Preoperative levels of NMN were found to be significantly elevated, while other blood test results did not show any significant abnormalities. The synthesis, secretion and release of CA are not completely dependent on the adrenal medulla, which may also be the reason why our patient had no typical symptoms despite the increase of NMN (11). Adrenal CT and PETCT imaging supported the diagnosis of PCC without evidence of lesions in other areas. The Endocrine Hypertension Working Group of the European Society for Hypertension recommends minimally invasive adrenalectomy as the preferred surgical approach for PCC, as it can minimize blood loss and shorten postoperative hospitalization (18). While guidelines suggest considering a cesarean section for PCC tumors larger than 5cm,as for our patient the PCC measures approximately 5cm in diameter. The surgical team observed only hepatic omentum tissue and some intestinal adhesions during the procedure, with no apparent abnormalities in the neighboring organs of the tumor. Consequently, they opted for laparoscopic right adrenal mass resection surgery. Surgical intervention has been shown to enhance overall survival (OS) (46). European guidelines suggest postoperative monitoring through blood tests, such as measurement of MN and NMN at 2–6 weeks post-surgery and annually thereafter, as well as imaging studies (CT/MRI) at 3 months, 6 months, and biennially thereafter (18). Another study proposes that patients with SDHA/B PPGL who are at a high risk of metastasis should consider undergoing biochemical tests every 6 to 12 months and imaging every 1 to 2 years (10). Following postoperative normalization of NMN levels, no recurrence or metastasis was detected five months post-operation for our patient. However, distant metastasis (lung, liver, bone) of PCC was discovered 16 months post-surgery. Imaging studies, including lung and liver CT, PETCT, and subsequent pathological examination of lung and bone metastases, provided compelling evidence of multiple site metastasis of PCC. The potential benefit of increasing the frequency of postoperative follow-up, such as every 6 months, for these patients is worth exploring. There is no standardized treatment for metastatic PPGL, but options include surgical resection, targeted radiolabeled carriers, thermal ablation, chemotherapy, and external irradiation (9). Chemotherapy, specifically with drugs like cyclophosphamide, vincristine, and dacarbazine, is preferred for advanced PPGL, especially in rapidly progressing cases. Tumors with mutations in the gene encoding Krebs cycle enzyme may respond better to temozolomide due to reduced expression of methylguanidine DNA methyltransferase (44, 47). Temozolomide chemotherapy was chosen based on the patient’s tolerance, however, due to significant side effects, treatment was discontinued after a single course. Octreotide and denosumab were administered subcutaneously starting at 16 months post-operation. The patient experienced intermittent hemoptysis during the advanced stage of lung metastasis, and TKIs were not utilized. Radionuclide therapy was deemed unsuitable due to the evident adverse effects observed during the relevant radionuclide examination. The patient had a low tolerance to certain treatments and a lower OS rate compared to previous reports on metastatic PPGL.

## 4 Conclusions

This study presents a case of metastatic PCC with uncommon DLST point mutations, characterized by high malignancy, rapid disease progression, and limited therapeutic efficacy. These cases warrant additional attention in determining the optimal timing for genetic sequencing, enhancing the frequency of monitoring, and developing personalized treatment strategies.

## Data availability statement

The original contributions presented in the study are included in the article/**Supplementary Material.** Further inquiries can be directed to the corresponding author.

## Ethics statement

The studies involving humans were approved by Ethics Committee of China-Japan Union Hospital of Jilin University. The studies were conducted in accordance with the local legislation and institutional requirements. The participants provided their written informed consent to participate in this study. Written informed consent was obtained from the individual(s) for the publication of any potentially identifiable images or data included in this article.

## Author contributions

CL: Conceptualization, Data curation, Formal Analysis, Funding acquisition, Validation, Writing – original draft. LH: Data curation, Resources, Writing – original draft. YS: Conceptualization, Formal Analysis, Project administration, Visualization, Writing – review & editing. RL: Formal Analysis, Funding acquisition, Validation, Visualization, Writing – review & editing.
