# Supplementary material for: Case report: A rare DLST mutation in patient with metastatic pheochromocytoma: clinical implications and management challenges
Source: Front Oncol. 2024 May 21;14:1394552. doi: 10.3389/fonc.2024.1394552 (PMC11148276; doi:10.3389/fonc.2024.1394552)
Supplement: Supplementary file 1 [file Table_1.docx]

Supplementary TABLE 1 Changes in CA, MN,NMN and NES in the patient.

| Time | MN（＜0.5nmol/L） | NMN（＜0.9nmol/L） | E（＜367pmol/L） | NE（＜2202pmol/L） | NSE（＜16.3ng/ml） |
| --- | --- | --- | --- | --- | --- |
| 2021.07 | 0.26 | ***3.62*** | ─ | ─ | 13.92 |
| 2021.07 | 0.24 | ***3.6*** | ─ | ─ | ─ |
| 2021.12 | ＜0.20 | 0.58 | ─ | ─ | 12.05 |
| 2022.11 | ＜0.08 | 0.16 | 183.9 | 2099.2 | 14.8 |
| 2023.03 | ─ | ─ | ─ | ─ | ***52.9*** |
| 2023.05 | ─ | ─ | ─ | ─ | 15.13 |
| 2023.07 | ─ | ─ | 36.7 | 441 | 15.13 |
| 2023.09 | 0.18 | ***6.01*** | 188.1 | ***6771.1*** | ***228.1*** |

MN,metanephrine;NMN,normetanephrine;E,epinephrine;NE,norepinephrine;NSE,neuron specific enolase.
