# Supplementary material for: Case report: A rare DLST mutation in patient with metastatic pheochromocytoma: clinical implications and management challenges
Source: Front Oncol. 2024 May 21;14:1394552. doi: 10.3389/fonc.2024.1394552 (PMC11148276; doi:10.3389/fonc.2024.1394552)
Supplement: Supplementary file 2 [file Table_2.docx]

Supplementary TABLE 2 Whole-exome Sequencing Report.

| Gene | Chromosomal location | Transcript exons | Nucleotide amino acid | Proband (female) | Normal frequency | Forecast | Pathogenicity analysis | Disease / phenotype | Source of variation |
| --- | --- | --- | --- | --- | --- | --- | --- | --- | --- |
| DLST | chr14:75356669 | NM_001933;  IVS6 | c.330+14A>G | het  21/43 | 0.0001 | - | VUS | PGL type 7  (OMIM:618475),AD | unknow |
| CCND1 | chr11:69462749 | NM_053056;  IVS3 | c.575- 13C>T | het 42/98 | 0.0001 | - | VUS | Von Hippel-Lindau syndrome (OMIM:193300),AD | unknow |

VUS, Variant of Uncertain Significance;AD, Autosomal Dominant.
