# Supplementary material for: Case report: A rare DLST mutation in patient with metastatic pheochromocytoma: clinical implications and management challenges
Source: Front Oncol. 2024 May 21;14:1394552. doi: 10.3389/fonc.2024.1394552 (PMC11148276; doi:10.3389/fonc.2024.1394552)
Supplement: Supplementary file 3 [file Table_3.docx]

Supplementary TABLE 3 Symptoms, signs, and treatment process.

| First visit and follow-up | Symptoms, signs, and treatment |
| --- | --- |
| First visit (2021.07) | The patient had intermittent abdominal pain for 1 month and visited the Department of Endocrinology and Urology. Normal vital signs and no abnormal findings in abdominal examination were noted. A right adrenal mass was found on CT scan, diagnosed as PCC. Preoperative preparation was done, followed by laparoscopic resection confirming the diagnosis. The patient recovered well postoperatively with stable vital signs. |
| Initial postoperative  follow-up (2021.12) | Normal blood pressure and heart rate, no recurrence or metastasis was detected. Continue regular monitoring. |
| The second postoperative  follow-up (2022.11) | PETCT revealed metastases of PCC in the lung, liver, and bone. Treatment includes temozolomide chemotherapy, octreotide somatostatin therapy, denosumab for bone metastasis, and monitoring vital signs and drug side effects. |
| The third postoperative  follow-up(2023.03） | Liver MRI and lung CT revealed enlarged and increased metastases. Temozolomide chemotherapy was stopped after one course due to severe side effects(unbearable nausea and vomiting). Octreotide and denosumab were continued along with traditional Chinese medicine anti-tumor treatment. |
| The fourth postoperative  follow-up（2023.05） | The lumbar spine MRI revealed more metastases, causing the patient's condition to worsen with decreased appetite, nausea, and vomiting. Treatment with octreotide and denosumab continued, and periodic hospital stays helped improve the patient's nutrition. |
| The fifth postoperative  follow-up（2023.07） | Liver and lung metastases are advancing quickly. The patient's condition deteriorated with symptoms of nausea, vomiting, and hemoptysis. Treatment with octreotide and denosumab continued, while adjustments were made to nutritional support, pain management, and other palliative care. |
| The sixth postoperative  follow-up（2023.09） | Lung metastases lead to pneumonia and respiratory failure, causing cachexia in the patient, which was the final stage of the tumor. Terminal care was provided to relieve the pain. |
