# Supplementary material for: Case report: A rare DLST mutation in patient with metastatic pheochromocytoma: clinical implications and management challenges
Source: Front Oncol. 2024 May 21;14:1394552. doi: 10.3389/fonc.2024.1394552 (PMC11148276; doi:10.3389/fonc.2024.1394552)
Supplement: Supplementary file 4 [file Table_4.docx]

Supplementary TABLE 4 The general laboratory test data.

| Laboratory tests | Laboratory sub projects | Results | Reference range |
| --- | --- | --- | --- |
| Blood routine analysis | White blood cell count  Red blood cell count  Hemoglobin count  Platelet count | 5.79 ×10^~^9/L  3.75 ×10^~^12/L  120.0 g/L  212 ×10^~^9/L | 4-10 ×10^~^9/L  3.5-5 ×10^~^12/L  120-160 g/L  100-300 ×10^~^9/L |
| Urinalysis | White blood cells  Red blood cells  Bacteria  Proteins  Glucose  Tubules | 24.9 /ul  2.70 /ul  43.20 /ul  Negative  Negative  0.0 /ul | 0-25 /ul  0-23 /ul  0-1200 /ul  Negative  Negative  0-1 /ul |
| Coagulation profile | Prothrombin time  Activated partial thromboplastin time  Thrombin time  Fibrinogen | 10.8 s  31.0 s  13.9 s  3.25 g/L | 9.4-12.5 s  25.4-38.4 s  10.3-16.6 s  2-4 g/L |
| Blood biochemistry | Alanine aminotransferase  Aspartate aminotransferase  Total protein  Albumin  Urea nitrogen  Creatinine  Blood sugar | 12.44 IU/L  14.92 IU/L  62.00 g/L  37.90 g/L  2.86 mmol/L  62.5 mmol/L  5.01 mmol/L | 5-40 IU/L  8-40 IU/L  62-83 g/L  35-52 g/L  2.5-6.1 mmol/L  46-92 mmol/L  3.9-6.1 mmol/L |
| Cortisol rhythms | Cortisol（7:00-9:00）  Cortisol（15:00-17：00）  Cortisol (23:00-1：00) | 18.2μg/dl  5.4 μg/dl  2.8 μg/dl | 4.3-24.9 μg/dl  2.9-17.3 μg/dl  0.0-6.7 μg/dl |
| Corticotropin rhythms | Corticotropin (7:00-9：00)  Corticotropin (15:00-17：00)  Corticotropin (23:00-1:00) | 39.02 pg/ml  10.54 pg/ml  6.46 pg/ml | 7.20-63.40 pg/ml  3.00-32.00 pg/ml  0.00-32.00 pg/ml |
| Hypertension three items  (lying position) | Cortisol (lying position)  Angiotensin II (lying position)  Aldosterone (lying position) | 7.10 pg/ml  117.41 pg/ml  219.4 pg/ml | 2.40-32.80 pg/ml  25.00-129.00 pg/ml  10.0-160.0 pg/ml |
| Hypertension three items  (orthostatic position) | Cortisol (orthostatic position)  Angiotensin II (orthostatic position)  Aldosterone (orthostatic position) | 37.44 pg/ml  106.54 pg/ml  451.5 pg/ml | 3.80-38.80 pg/ml  49.00-252.00 pg/ml  40-310 pg/ml |
| 24-hour urinary free cortisol test | - | 66.19 μg/24 hours | 50.00-437.00 μg/24 hours |
